# A Small Force Remodels a Large Protein to Unleash Its Deadly Potential

**DOI:** 10.1371/journal.pbio.1001486

**Published:** 2013-02-19

**Authors:** Richard Robinson

**Affiliations:** Freelance Science Writer, Sherborn, Massachusetts, United States of America

**Figure:**
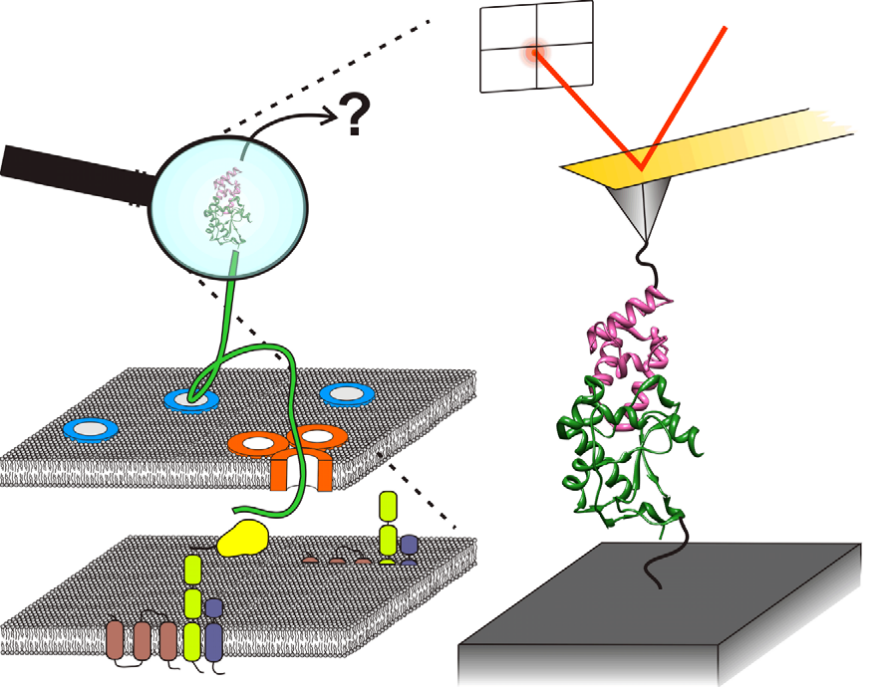
*E. coli* protects itself from colicin E9, a protein secreted to kill related strains, by co-expressing a second protein, Im9, which binds to and inactivates E9. Cells apply a small force to dissociate the E9:Im9 complex when E9 activation is required to attack neighboring strains. Image credit: Oliver Farrance.

Poisons are powerful weapons, but they carry an inherent risk for those who wield them—becoming one's own victim. *E. coli* bacteria, which manufacture a DNA-destroying enzyme used to kill other cells, have solved this problem by blocking part of the enzyme's surface with a second protein while it awaits delivery. The two bind so tightly, in fact, that researchers have had trouble figuring out how the cell manages to ever separate them when the time comes. In a new study in *PLOS Biology*, Oliver Farrance, David Brockwell, and colleagues show that a small applied force can greatly reduce the underlying stability of the complex and vastly increase the dissociation rate.

The enzyme, called colicin E9, has three segments, the first two of which are responsible for binding to a receptor on the target cell and translocating the enzyme through the target membrane. The third segment cleaves DNA in the target cell, and it is this toxic portion of colicin, termed the C domain, that must be kept in an inactive form until the protein is released. The bacterium accomplishes this with an “immunity” protein called Im9, which binds near the active site. The two bind so avidly that the lifetime of the complex is measured in days. Yet, they dissociate within minutes during an attack on a neighboring bacterium.

This rapid and profound change in dissociation rate of the two proteins in the absence of a large input of energy suggested to the authors that some more subtle and specific change in the system was likely at work that altered the underlying energy landscape. To test that proposition, they turned to atomic force microscopy (AFM). By attaching the colicin molecule to a fixed substrate, and attaching the immunity protein molecule to the probe of the AFM microscope, they could pull them apart, and measure the force required to separate the two.

They found that the affinity of the two proteins changed dramatically once a small force was applied. The change in affinity was such that the rate of dissociation was reduced from days to milliseconds, well within the timescale of intoxication. These results suggested that the small applied force, whatever its biological source might be, was sufficient to destabilize the complex, a phenomenon the authors termed a “trip bond.” They next explored the mechanism of the trip bond, by mutating various residues in the C domain, and by pairing the C domain with other, less avidly binding immunity proteins. Taken together, their results showed that the small applied force likely acts to partially remodel or unfold a segment of the colicin protein, and that this relatively minor structural change significantly alters the surface that binds the immunity protein, causing a collapse in the affinity of the interaction. Support for that model was strengthened when the authors introduced one or more covalent disulfide bonds into the structure, physically preventing the remodeling and drastically increasing the force required for dissociation.

Exactly how the bacterium itself applies this small force is not yet clear, but the necessary force, about 20 picoNewtons, is in the range of what could be supplied by a variety of cellular phenomena, including a molecular motor or a proton gradient, both known to be present in *E. coli*. The ability of a small force to remodel the energy landscape of a large protein is likely at work in many other proteins as well, and may explain a variety of control mechanisms in both bacteria and eukaryotes.

**Farrance OE, Hann E, Kaminska R, Housden NG, Derrington SR, et al. (2013) A Force-Activated Trip Switch Triggers Rapid Dissociation of a Colicin from Its Immunity Protein. doi:10.1371/journal.pbio.1001489**

